# Pulmonary Fibroblast Heterogeneity and Functional Remodeling in Idiopathic Pulmonary Fibrosis: State Persistence and Reversibility

**DOI:** 10.3390/ijms272110031

**Published:** 2026-11-09

**Authors:** Lincha Tian, Xinru Feng, Bo Xiao, Lixia Hou, Xiulin Yi, Manjie Chen, Biwen Mo, Dong Yao

**Affiliations:** 1 Department of Respiratory and Critical Care Medicine, The Second Affiliated Hospital of Guilin Medical University, Guilin 541199, China; 15177626173@163.com (L.T.); fxin_ru@163.com (X.F.); 15577368665@163.com (X.Y.); c2001mj@163.com (M.C.); 2 The Laboratory of Respiratory Disease, The Affiliated Hospital of Guilin Medical University, Guilin 541001, China; boxiao@glmu.edu.cn (B.X.); houlixia_03@163.com (L.H.); 3 The Key Laboratory of Respiratory Diseases (Guilin Medical University), Education Department of Guangxi Zhuang Autonomous Region, Guilin 541001, China; 4 Guangxi Key Laboratory of Metabolic Reprogramming and Intelligent Medical Engineering for Chronic Diseases, Guilin Medical University, Guilin 541199, China; 5 Guangxi Clinical Research Center for Diabetes and Metabolic Diseases, Guangxi Health Commission Key Laboratory of Glucose and Lipid Metabolism Disorders, The Second Affiliated Hospital of Guilin Medical University, Guilin 541199, China; 6 School of Artificial Intelligence in Medicine, Guilin Medical University, Guilin 541199, China

**Keywords:** pulmonary fibrosis, idiopathic pulmonary fibrosis, lung fibroblasts, cellular heterogeneity, functional remodeling, targeted strategies

## Abstract

Pulmonary fibrosis is a common pathological endpoint of several interstitial lung diseases. Idiopathic pulmonary fibrosis (IPF), in particular, has an unknown cause, produces progressive scarring of the lung parenchyma, and carries a poor prognosis. Single-cell and spatial omics studies have shown that lung fibroblasts comprise multiple populations with distinct anatomical locations and functional states. However, how these states are established, interconverted, and maintained in the injured microenvironment remains incompletely understood and has not been directly validated. This review uses IPF as the primary clinical context and draws complementary mechanistic evidence from other fibrotic lung diseases and animal models. It examines fibroblast origins and state heterogeneity; signaling networks involving transforming growth factor-β (TGF-β), Wnt, and Yes-associated protein (YAP); interactions among epithelial cells, immune cells, and fibroblasts; and epigenetic and metabolic remodeling. On this basis, we propose a two-layer stability model of epigenetic maintenance and signaling feedback as a working framework for understanding the persistence of profibrotic states. We also discuss how treatment might move from nonselective inhibition toward restricting pathological states, eliminating pathogenic subpopulations, and preserving reparative function. The causal sequence proposed by this model, the necessity of its components, and the thresholds for state reversal remain unresolved and require testing through lineage tracing, assessment of phenotypic stability after stimulus withdrawal, and sequential combination interventions.

## 1. Introduction

Idiopathic pulmonary fibrosis (IPF) is a progressive fibrosing interstitial pneumonia of unknown cause. Pulmonary fibrosis is also a shared pathological endpoint of other interstitial lung diseases, including connective tissue disease-associated interstitial lung disease (ILD), occupational and environmental lung disease, and drug-induced lung injury. In IPF, excessive extracellular matrix (ECM) deposition destroys alveolar architecture and impairs lung function [1]. Lung fibroblasts and their myofibroblast derivatives are central effectors of ECM synthesis and remodeling [2]. Single-cell and spatial omics have shown that lung fibroblasts are not a homogeneous population; they form a dynamic community of anatomically distinct subsets and disease-associated states. Their composition must therefore be interpreted in disease context.

Fibroblast heterogeneity reflects differences in anatomical location, transcriptional state, and response to injury. CTHRC1^+^ fibroblasts accumulate in fibrotic lesions and show high expression of collagen-related genes [3]. In experimental models, lipofibroblasts and Ebf1^+^ cells undergo state transitions and contribute to matrix remodeling [4], whereas Meflin^+^ cells appear protective [5]. GREM1/PPP2R3A-associated states may contribute to early fibrosis in some models [6]. Together, these observations indicate that multiple fibroblast states contribute to fibrosis, although their nomenclature, lineage relationships, and relative importance in human disease remain unresolved. Phenotype is similarly fluid: an individual subset may change function across disease stages, and different subsets may enter the same functional state. A population label should not, therefore, be treated as a fixed cell fate.

Here, functional remodeling refers to changes in fibroblast transcriptional, epigenetic, metabolic, and secretory states, together with the resulting changes in migration, contraction, and ECM synthesis. We use IPF as the primary clinical context and draw supporting mechanistic evidence from other fibrotic lung diseases and animal models, noting the limits of extrapolation where relevant.

The review focuses on fibroblast origin, state heterogeneity, and the transition from quiescence to activation. It integrates signaling, epigenetic and metabolic regulation, intercellular interactions, model limitations, and targeted strategies within a single conceptual framework. The two-layer stability model is intended to organize evidence, not to serve as an established law of disease. To avoid conflating transcriptional clusters with stable cell types, we consider lineage origin, spatial location, disease stage, and functional validation, and we distinguish three evidence levels: associations in human tissue, animal lineage tracing, and in vitro mechanisms. This approach clarifies how heterogeneity shapes functional remodeling and why evidence strength constrains therapeutic extrapolation.

The review is organized as follows. Section 2 addresses fibroblast origins and state heterogeneity, and Section 3 examines functional remodeling. Section 4, Section 5 and Section 6 discuss signaling pathways, intercellular interactions, and epigenetic and metabolic regulation. Section 7 evaluates experimental models, Section 8 examines therapeutic strategies, Section 9 considers translational challenges and future directions, and Section 10 presents the conclusions.

## 2. Origins and State Heterogeneity of Lung Fibroblasts

### 2.1. Origins and Lineage Relationships of Lung Fibroblasts

**Figure 1:**
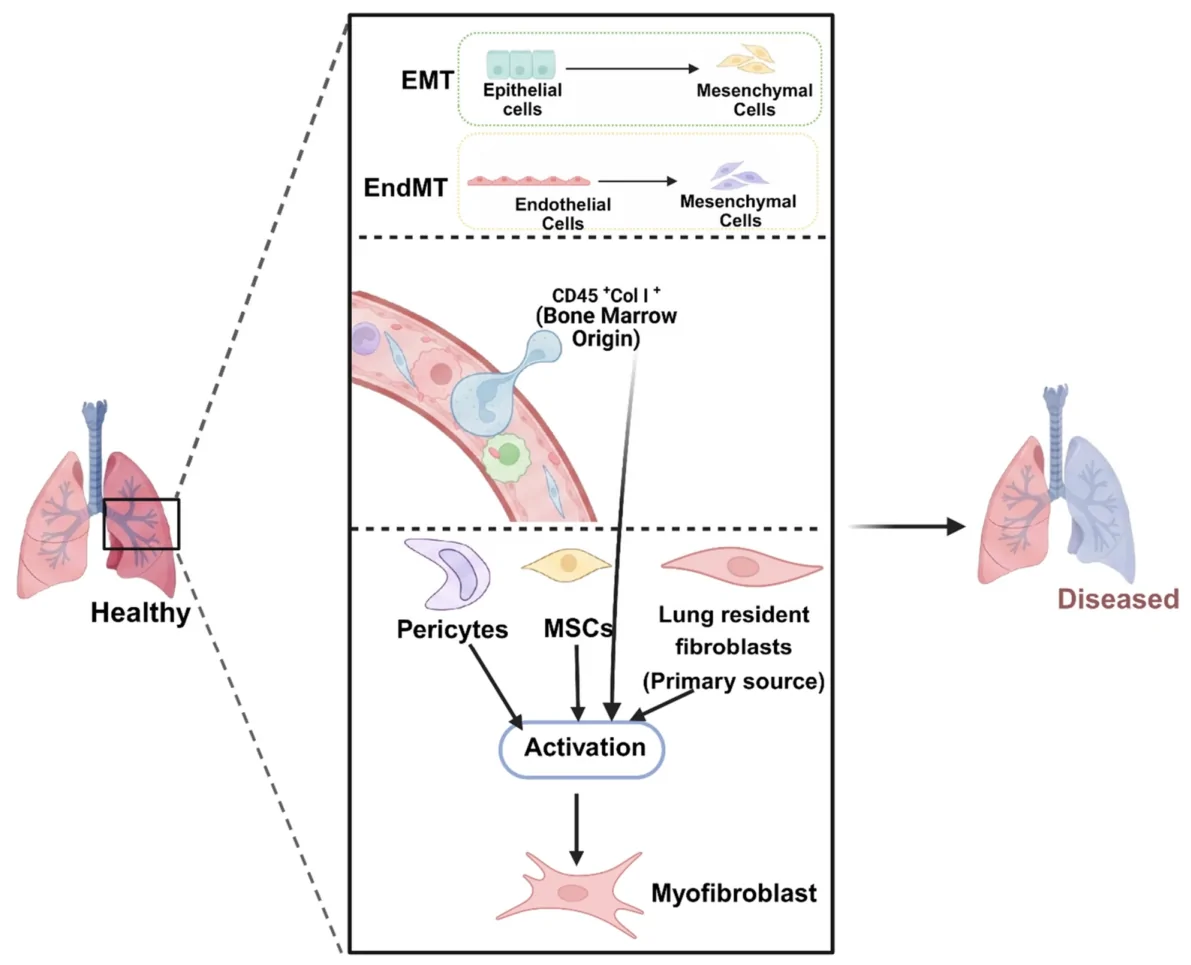
Origins of myofibroblasts and evidentiary boundaries in pulmonary fibrosis. Resident intrapulmonary fibroblasts are the principal source of pathological matrix-producing cells; pericytes, circulating fibrocytes, and mesenchymal stromal cells may contribute to profibrotic states in specific models. Epithelial and endothelial cells can acquire partial mesenchymal programs and paracrine activity, but evidence that they directly generate large numbers of stable myofibroblasts remains insufficient.

Resident intrapulmonary mesenchymal cells are currently the best-supported source of myofibroblasts in fibrotic lung lesions, although evidence differs among the proposed sources. Figure 1 summarizes these origins and the strength of the supporting evidence. Single-cell and lineage-tracing studies in mice show that alveolar and adventitial fibroblasts expand, migrate, and acquire pathological high-ECM states [3,7]. Recent fate-mapping and transition studies further link alveolar fibroblasts to injury-associated inflammation and fibrosis and identify a RUNX2-associated alveolar-to-pathological transition; these studies provide temporal evidence but do not quantify the human contribution. Human single-cell studies by Adams and Habermann characterize disease-associated epithelial and mesenchymal populations, while Buechler et al. provide cross-tissue context for fibroblast lineages [8,9,10,11,12]. Epithelial and endothelial cells can acquire partial mesenchymal programs and paracrine activities during epithelial–mesenchymal transition (EMT) and endothelial–mesenchymal transition (EndMT); however, evidence that they generate large, stable myofibroblast populations remains controversial [13,14,15,16]. In a classical bleomycin lineage-tracing study, labeled epithelial cells did not become myofibroblasts [17], arguing against designating EMT as the principal source of myofibroblasts in human IPF. Circulating fibrocytes, pericytes, and mesenchymal stromal cells may also contribute to inflammation, matrix regulation, and repair in specific models, but their contribution to the long-term collagen-producing pool varies with the model, labeling strategy, and observation period [18,19]. Classical studies reported CXCL12-dependent fibrocyte recruitment in bleomycin-injured mice and fibrocytes in IPF lung tissue [20,21], whereas resident-cell tracing supports an intrapulmonary source [17]; their relative contributions therefore remain model- and method-dependent. Resident mesenchyme itself includes regionally distinct alveolar, peribronchial, and adventitial populations with different proliferative, migratory, and ECM-synthetic capacities. Claims about cellular origin should specify the species, model, lineage tools, and functional endpoint; marker co-expression alone is insufficient to establish lineage conversion.

### 2.2. Fibroblast Subpopulations in Healthy and Fibrotic States

**Figure 2:**
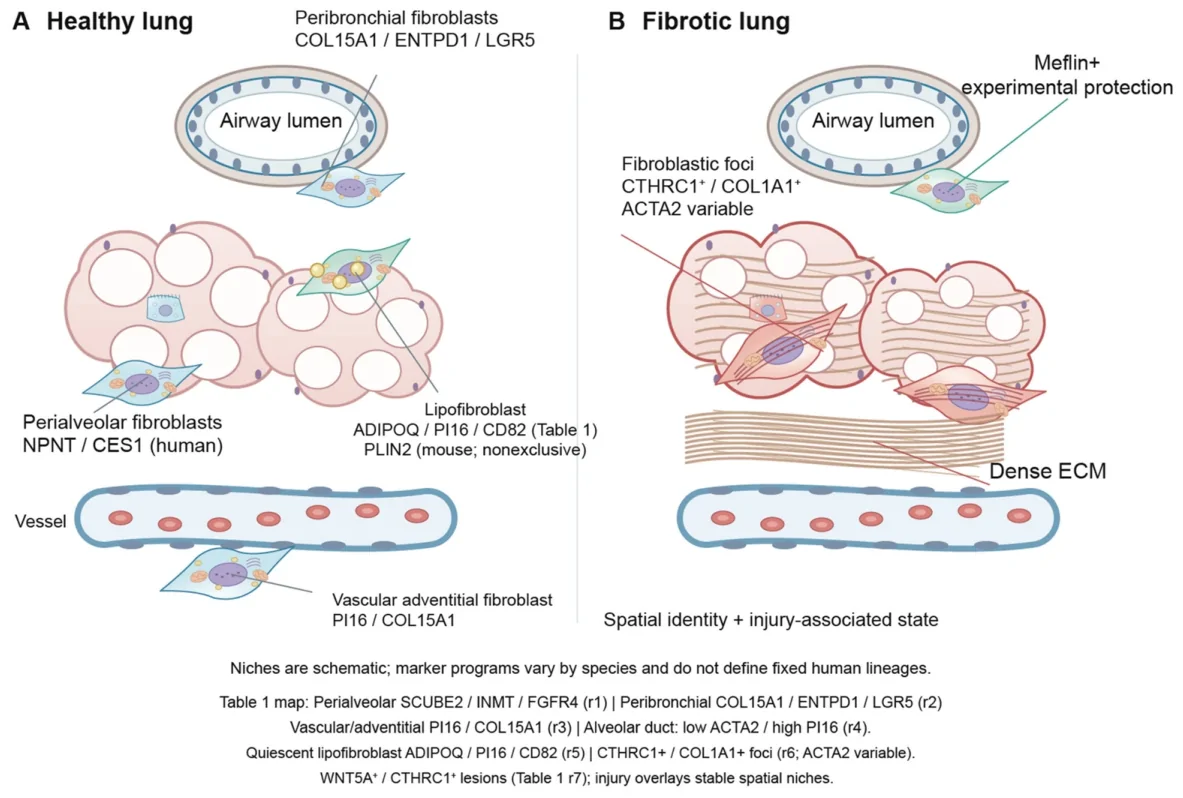
Spatial organization of fibroblast niches in (**A**) healthy and (**B**) fibrotic lungs. The diagram distinguishes perialveolar, peribronchial, vascular-adventitial, alveolar-duct, and lipofibroblast programs and links selected markers to Table 1 (row numbering excludes the header). Fibrotic foci are annotated as CTHRC1^+^/COL1A1^+^ with variable ACTA2, and WNT5A^+^/CTHRC1^+^ lesions are shown as injury-associated states. PLIN2 is a non-exclusive mouse marker. Locations and marker programs are schematic and species-dependent; they do not define fixed human lineages. Protective effects of Meflin are experimental.Colors distinguish schematic cell states and anatomical compartments.

Healthy lung fibroblasts can be classified by anatomical location and transcriptional profile into alveolar, peribronchial, adventitial, and other populations, some of which have lipid-metabolism-related features. In fibrosis, these populations expand, migrate, and alter their transcriptional programs, while high-ECM states such as CTHRC1^+^ fibroblasts appear [3,4,22]. Meflin^+^ fibroblasts are protective in experimental models [5], but their abundance, stability, and therapeutic accessibility in human IPF remain uncertain. Fibrosis therefore appears to involve dynamic rearrangement across anatomical subsets and disease-associated states rather than a simple shift between two fixed cell types. In the healthy lung, alveolar fibroblasts maintain the alveolar epithelial niche and matrix turnover, whereas adventitial fibroblasts surround vessels and airways. Lipid-metabolism-related fibroblasts can provide metabolic and paracrine support to alveolar type 2 epithelial cells (AEC2). Table 1 and Figure 2 summarize the molecular features, supporting evidence, anatomical niches, and disease-associated shifts in the principal populations.

### 2.3. Resolving Heterogeneity with Single-Cell and Spatial Omics

Single-cell transcriptomics, spatial omics, and epigenomic methods have increased the resolution of fibroblast heterogeneity, but they do not by themselves provide a stable classification. Cluster number varies with sampling site, species, disease stage, dissociation method, and clustering parameters; counts should therefore not be treated as a universal standard. More reproducible observations include alveolar, peribronchial, and adventitial populations and a disease-associated CTHRC1^+^ state enriched in fibrotic lesions [23,28,29]. ATAC-seq and other epigenomic studies link regulatory networks involving AP-1, RUNX, and EGR, together with altered chromatin accessibility, to sustained activation [30,31]. These data suggest that epigenetic state may help maintain profibrotic phenotypes, but they do not show that such states are irreversible in vivo. Spatial transcriptomics adds tissue context, and multi-omic integration can identify candidate regulatory elements and transcription-factor networks. At the same time, dissociation bias, ambient RNA, doublets, and batch effects can create false clusters. Reliable nomenclature therefore requires histological localization, protein validation, independent replication, and, when possible, functional evidence.

## 3. Functional Remodeling of Lung Fibroblasts from Homeostasis to Profibrotic States

### 3.1. Major Features of Homeostatic Fibroblasts

Homeostatic lung fibroblasts show relatively low contractile and ECM-synthetic activity while maintaining matrix turnover, the epithelial niche, and lipid metabolism [32]. PI16 has been supported by mouse single-cell and spatial data and by functional testing in human lung slices [24]; CD82 marks a lipofibroblast subset in human single-cell, tissue, and flow-cytometric analyses [4]; and CD36 and CD97 have been assessed by immunofluorescence and flow cytometry in primary human fibroblasts and IPF tissue [25]. VCAM1/CD106 is excluded because it is mesothelial-associated in the cited human data and low or nonspecific in mouse fibroblast datasets [4,25]. These markers do not define a universal quiescent-fibroblast signature. Instead, homeostasis and profibrotic activation lie on a continuous, plastic spectrum. Intrinsic factors such as KLF4, calcium signaling, and the TGF-β response threshold influence activation propensity [33,34], and some lipofibroblast-associated states can shift into inflammatory and myofibroblast-like states in experimental models and revert as injury resolves [35]. Fibroblasts are not passive structural cells: they continually renew collagen, elastic fibers, and proteoglycans, regulate matrix mechanics, and support epithelial repair through Wnt, FGF, BMP, and lipid signaling. Quiescent and homeostatic are therefore relative terms, and fibroblasts in different regions retain basal metabolic and matrix-turnover activity. Because most markers derive from mouse lineage studies or in vitro sorting and overlap in the human lung, defining a homeostatic population should require multiple markers, spatial context, and functional validation rather than a single surface molecule.

### 3.2. Fibroblast Activation and Myofibroblast Formation

Fibroblast activation is initiated by a combination of injury, inflammation, and matrix stiffening. Early changes include increased proliferation and migration, induction of profibrotic genes, and altered secretion. Some cells then enter a myofibroblast differentiation trajectory that is sustained by both ongoing signaling and matrix conditions. Matrix stiffening and TGF-β signaling can reinforce each other during early activation, although the timing and necessity of this feedback remain unresolved.

Sustained activation promotes a myofibroblast-like state with increased contractile and ECM-synthetic capacity. α-SMA stress fibers, COL1A1, and CTHRC1 vary by subset, location, and disease stage, and no single marker identifies all pathological fibroblasts [36,37]. Myofibroblasts are therefore best viewed as an important disease-associated state rather than an irreversible terminal lineage. The ECM they produce increases stiffness and activates mechanosignaling [38,39]. Early in repair, this response restores tissue integrity. With persistent injury, unresolved inflammation, or continued matrix stiffening, TGF-β, integrin, and YAP/TAZ signaling can reinforce one another and convert a transient reparative response into persistent scarring. Contractility, cross-linking, and release of active TGF-β can further modify the niche and establish a self-reinforcing relationship between cell state and matrix properties [38,39,40].

### 3.3. Major Phenotypes of Functional Remodeling

When fibroblasts transition from quiescence to activation, functional remodeling proceeds along several overlapping dimensions, and the combination depends on cell origin, disease stage, and stimulus. The contractile apparatus strengthens, and some cells assemble α-SMA stress fibers and generate greater force [37]. ECM synthesis and remodeling increase, with corresponding changes in type I/III collagen, fibronectin, and related enzymes [4,39]. In certain subsets and models, proliferation, migration, or invasion also increases, promoting accumulation at sites of injury [41].

**Figure 3:**
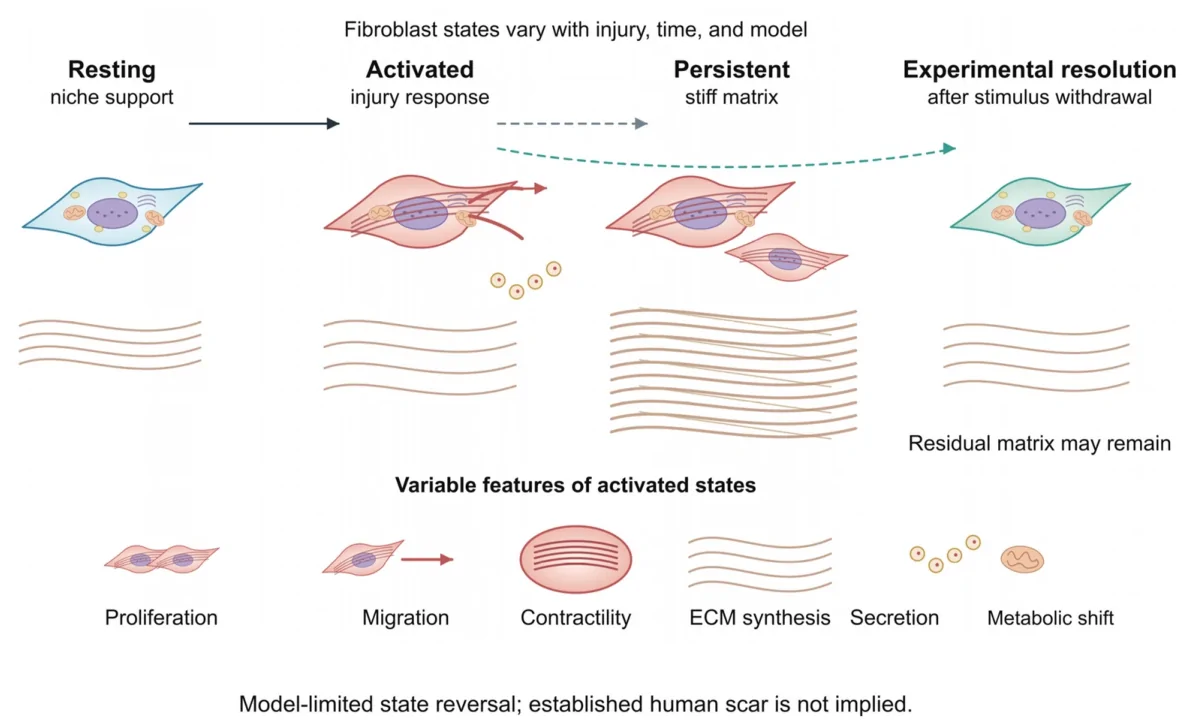
Functional states of lung fibroblasts in relation to cell morphology and matrix context. Resting support can shift toward injury-associated activation, with persistence and experimental resolution represented as alternative, context-dependent trajectories. The six functions shown are proliferation, migration, contractility, ECM synthesis, secretion, and metabolic reprogramming; they do not necessarily occur together. Dashed transitions indicate model-limited possibilities and do not imply reversal of established human scarring. Solid arrows show activation or progression; dashed arrows show candidate resolution trajectories. Colors distinguish the illustrated cell states.

The secretory profile shifts toward CTGF, PDGF, IL-11, and chemokines, which can act on fibroblasts, epithelial cells, and immune cells through autocrine or paracrine signaling [42,43,44]. Glycolysis, glutamine utilization, and mitochondrial function are also reprogrammed in some activated states, providing energy and biosynthetic support [45,46,47,48,49,50,51,52]. These features do not appear synchronously in all cells, and no single marker defines a fixed pathogenic subset. Their causal importance in human IPF must be established through spatial localization, longitudinal observation, and cell-specific perturbation. Figure 3 summarizes these overlapping trajectories.

### 3.4. Persistence and Reversibility of Profibrotic States

Scarring irreversibility should be distinguished from fibroblast-state irreversibility. Established structural fibrosis in IPF is generally difficult to reverse, whereas some activated fibroblast states can revert after intervention or injury resolution [33,35,53]. Chromatin accessibility, DNA methylation, histone modifications, persistent signaling, and ECM stiffening may all increase the threshold for reversal [30,31,54], but no longitudinal study has shown that a particular fibroblast state is absolutely irreversible in vivo. Stable maintenance therefore means that profibrotic features persist after removal of the initiating stimulus; it does not imply that the state cannot be reprogrammed. Persistence can reflect ongoing external stimuli, including recurrent epithelial injury, abnormal immune signaling, and matrix stiffening, or intracellular memory, including stable chromatin changes, altered DNA methylation, metabolic dependence, and autocrine circuits. Evidence from post-withdrawal culture, transplantation, and in vivo lineage tracing is complementary, and none of these approaches alone establishes true reversal. Functional reversal should therefore be assessed by durable improvement in ECM production, tissue mechanics, and lung function after treatment withdrawal, not by a single marker.

### 3.5. cAMP-Dependent Modulation of Fibroblast States

Cyclic AMP (cAMP) is a key inhibitory signal in fibroblast activation and may create conditions permissive for state reversal. Protein kinase A downstream of cAMP reduces RhoA/ROCK-dependent actomyosin contractility and MRTF/SRF-dependent transcription, thereby suppressing the contractile and matrix-producing program of myofibroblasts. In some contexts, cAMP signaling also limits nuclear YAP/TAZ activity, linking it to the mechanically maintained profibrotic state. These effects support a state-modulation strategy, but they do not establish that established human scarring can be reversed.

Pharmacological evidence is consistent with this hypothesis. Treprostinil, acting through prostacyclin receptor-mediated cAMP signaling, reduced fibroblast proliferation and ECM deposition, whereas preferential PDE4B inhibition with nerandomilast reduced contractility and induced a transcriptional profile consistent with dedifferentiation in cultured human IPF fibroblasts [55,56]. The nerandomilast findings are notable because PDE4 inhibition raises cAMP and may combine antifibrotic and immunomodulatory activity. Nevertheless, most evidence comes from cultured human cells or preclinical models. Future studies should determine whether these effects persist after drug withdrawal and whether they improve tissue mechanics, alveolar repair, or lung function, rather than only a single activation marker.

## 4. Major Signaling Pathways in Fibroblast Functional Remodeling

**Figure 4:**
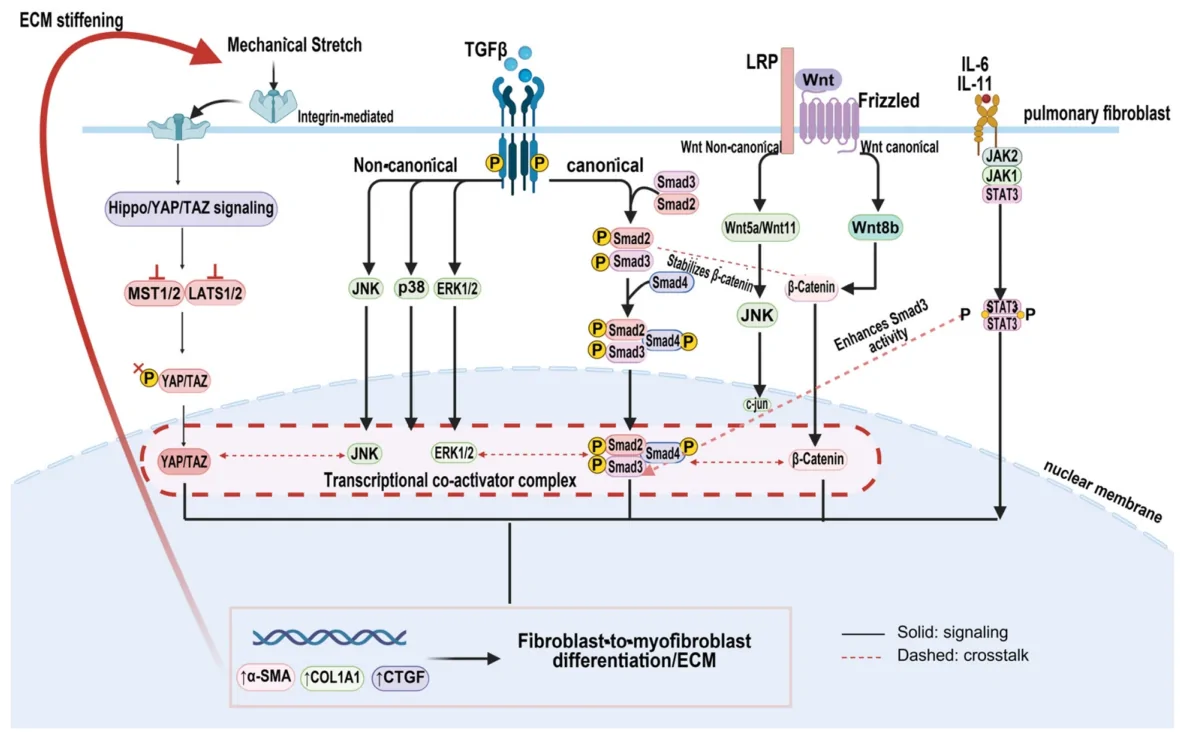
Convergence and feedback among fibroblast signaling pathways. TGF-β/Smad, Wnt/β-catenin, integrin-FAK-YAP/TAZ, and cytokine pathways can converge on overlapping profibrotic programs in specific cells and models, but they do not necessarily form a single physical complex. IL-6/IL-11-JAK/STAT3 is distinct from IL-13-STAT6; dashed lines indicate context- and model-dependent crosstalk. YAP/TAZ denotes the activated nuclear state, and phosphorylation symbols are not used as nonspecific indicators of activation. Solid lines indicate signaling connections; dashed lines indicate crosstalk. Arrowheads show the direction of the illustrated signaling relationship.

Fibroblast remodeling is shaped by multiple intersecting signals, not by a single dominant pathway. TGF-β/Smad, Wnt/β-catenin, integrin-FAK-YAP/TAZ, and cytokine pathways act in different cell populations and models and converge on partially overlapping transcriptional and functional programs. Existing evidence supports pathway redundancy and ECM feedback, but it does not show that all patients or disease stages share the same degree of synergy. When evaluating mechanisms or therapeutic targets, direct molecular interactions, transcriptional convergence, and shared phenotypic outputs should be distinguished (see Figure 4).

### 4.1. TGF-β/Smad Signaling

Transforming growth factor-β (TGF-β) signaling is one of the best-supported regulatory axes in studies of lung fibroblast activation. After TGF-β1 binds the TβRII/TβRI receptor complex, it can induce Smad2/3 phosphorylation and, together with Smad4, regulate fibrosis-related genes such as COL1A1 and ACTA2; it can also influence cell migration, survival, and ECM synthesis through non-Smad branches including ERK, JNK, and p38 MAPK [42,57,58]. This axis is enhanced in human IPF tissues, primary cells, and multiple animal models, although the magnitude of enhancement, cellular source, and temporal appearance are not fully consistent. TGF-β should therefore be regarded as an important upstream input rather than the sole input, and its activation level alone cannot establish that cell fate has become irreversible.

Whether TGF-β signaling persists depends on the combined regulation of receptor stability, negative-feedback factors, and the ECM environment. Smad7, Ski/SnoN, and related factors can limit signal propagation; in experimental studies, LMO7 promotes Smad7 degradation and enhances TβRI stability, thereby increasing the TGF-β response [59]. DDR2 is a collagen-activated receptor tyrosine kinase, and related findings indicate that DDR2 signaling can cooperate with the TGF-β/Smad program, but current evidence is insufficient to characterize DDR2 as a direct inducer of TGF-β upregulation in all pulmonary-fibrosis contexts [60]. RUNX1, MYH9, BMP4, and LTBP2 can also regulate differentiation or TGF-β-related outputs in specific systems [36,37,41,61]. These findings show that the pathway contains both context-dependent amplification and braking mechanisms; its necessity, druggability, and therapeutic window in human IPF still require testing in cell-type-specific studies.

### 4.2. Wnt/β-Catenin Signaling

Wnt signaling includes both the canonical β-catenin-dependent pathway and multiple noncanonical branches and cannot be reduced to a single axis with a uniform direction of effect. In models of resident pulmonary mesenchymal cells, Wnt8b can promote myofibroblast-like differentiation, whereas noncanonical ligands such as Wnt5a and Wnt11 can alter cell state through pathways including JNK/c-Jun [62,63]. After canonical Wnt activation, β-catenin is stabilized and enters the nucleus, where it regulates target genes such as AXIN2 together with TCF/LEF; however, readouts such as MMP7 may also originate from epithelial cells, so whole-lung expression alone cannot establish a direct effect within fibroblasts [64]. The sources, receptor combinations, and timing of action of different Wnt ligands may vary, and this contextual variation is a prerequisite for interpreting results across models. In a murine sclerodermatous cGvHD model, the PORCN inhibitor GNF6231 reduced associated pulmonary fibrosis; this model-specific result does not establish efficacy in IPF [65].

In some experimental systems, Wnt/β-catenin and TGF-β signaling reinforce one another; for example, TGF-β1 can affect β-catenin stability through ACP5 and promote fibroblast activation [66]. Wnt signaling also participates in the maintenance and differentiation of alveolar epithelial progenitors, so inhibiting this pathway may concomitantly affect repair [67]. In a silica-induced model, inhibition of Wnt/β-catenin reduced EMT-associated markers and the extent of fibrosis [68], but this result cannot directly establish that epithelial cells undergo complete lineage conversion in human IPF. ICG-001 and HSP90 inhibitors have shown antifibrotic activity in preclinical studies [64,69]; clinical efficacy, long-term safety, and cell selectivity still require further validation.

### 4.3. Integrin–FAK–YAP/TAZ Mechanotransduction Axis

ECM stiffening is both a consequence of disease and a continuing mechanical input during pulmonary-fibrosis progression. Fibroblasts sense matrix properties through integrins, focal adhesions, and the cytoskeleton, thereby initiating signals such as FAK/Src and altering migration, contraction, and ECM synthesis. Multiple integrin subtypes may participate in mechanosensing and cell invasion; α6β1 integrin is associated with the regulation of matrix-stiffness-dependent invasion by pulmonary myofibroblasts [70,71,72]. Because the ligands and functions of individual integrins differ, the complete mechanosensing process cannot be summarized as a single subtype switch.

Multiple mechanical inputs may ultimately converge on the Hippo/YAP/TAZ pathway. ECM stiffening alters Hippo pathway activity through integrins, the cytoskeleton, and focal adhesions; weakens some LATS-dependent inhibition; and promotes nuclear accumulation of YAP/TAZ, which together with TEAD transcription factors drives expression of profibrotic and pro-proliferative genes [73]. Pulmonary-fibrosis models show dysregulated Hippo signaling and high YAP expression; inhibiting YAP nuclear localization markedly reduces collagen deposition, supporting the interpretation that mechanical signals promote fibrosis through the YAP/TAZ axis [74]. Mechanosensitive ion channels such as Piezo2 may also convert matrix mechanical features into cell-activation signals [39].

YAP/TAZ can cooperate with Smad- and β-catenin-related transcriptional programs, allowing mechanical inputs to converge transcriptionally with TGF-β and Wnt signaling [75,76]. It can therefore be studied as a regulatory node integrating multiple pathways; however, its physiological functions are broad, and cell-type differences and potential toxicity mean that it cannot currently be described simply as an ideal target.

### 4.4. Other Relevant Pathways

Cytokine pathways must be distinguished according to the specific ligand and STAT member involved. In specific systems, IL-6 and IL-11 mainly affect fibroblast activation and metabolism through JAK/STAT3 and related branches [42,43,44,77]; IL-13 is more commonly associated with JAK/STAT6 and should not be subsumed into a unified JAK2/STAT3 axis. PDGF and FGF mainly promote cell proliferation, migration, or survival through receptor tyrosine kinases and pathways including MAPK and PI3K–Akt. JAK inhibitors such as baricitinib and ruxolitinib show activity in pulmonary-fibrosis or related fibrotic models, but this is insufficient to establish their use for IPF treatment [78,79,80]. MAPK, PI3K–Akt–mTOR, and NF-κB can also connect stress, metabolic, and inflammatory inputs, while AMPK is protective in some models [81]. The dominant pathway varies with cell state, stimulus source, and disease stage. The cGAS–STING pathway is another candidate inflammatory signal in pulmonary fibrosis; fluvoxamine-associated pathway modulation has been reported in a bleomycin model, but this does not establish fibroblast-specific causality or efficacy in human IPF [82].

### 4.5. Signaling Crosstalk and Feedback Networks

TGF-β/Smad, Wnt/β-catenin, YAP/TAZ, JAK/STAT, and MAPK pathways intersect at several levels. TGF-β and Wnt can regulate each other [83]; matrix stiffness can activate YAP/TAZ through integrin–FAK signaling and converge functionally with Smad or β-catenin programs [75,76]; and JAK/STAT3 and p38 MAPK can modulate TGF-β responses [84,85]. Such redundancy and feedback do not imply that these factors form a fixed physical complex or that blocking one node will inevitably be compensated by another. We refer to this functional convergence and ECM feedback collectively as a signaling feedback network. Its necessity, temporal organization, and reversal threshold remain to be established through combinatorial perturbation, post-withdrawal observation, and cell-type-specific experiments. Human studies mainly demonstrate disease-associated pathway expression, whereas causal and target-modulation evidence for IL-11, JAK/STAT, and YAP/TAZ is still predominantly experimental; none of these pathways has been shown to be necessary in human IPF [42,43,44,75,76,77,78,79,80].

## 5. Regulation of Fibroblast Function by Intercellular Interactions

### 5.1. Fibroblasts and the Local Microenvironment

Lung fibroblasts produce and remodel ECM; secrete growth factors, chemokines, and extracellular vesicles; and sense matrix stiffness. Single-cell studies reveal differences among anatomical subsets and disease states in ECM, chemokine, and SASP programs [86]. Some high-ECM or inflammatory states may amplify the profibrotic niche, but establishing a core driver population requires lineage tracing, cell-specific perturbation, and spatial validation in human tissue. MMP-TIMP imbalance, adhesion molecules, and SASP are all associated with matrix remodeling [4,87], although their relative causal contributions vary across disease causes and stages.

MMPs and their inhibitors are often imbalanced in pulmonary fibrosis. TIMP1 is elevated in some human samples and models and can alter MMP activity and ECM turnover, but its effects are not uniformly profibrotic or antifibrotic [87]. Some profibrotic states co-express adhesion molecules, chemokines, and matrix regulators and may influence immune-cell localization; this causal relationship requires cell-specific testing [4]. Senescent fibroblasts may contribute through SASP and ECM imbalance [28,86], but associations with progression or prognosis require validation in independent cohorts [88].

Fibroblasts also shape immune-cell recruitment through mediators such as CXCL12 and CCL2. In a humanized IPF model, PD-L1-high invasive fibroblasts promoted fibrosis, whereas in a JUN-driven scleroderma model, CD47/PD-L1 signaling was linked to clearance of abnormal fibroblasts [89,90]. These observations raise the possibility of checkpoint-like regulation of mesenchymal persistence [91], but differences in disease context and cell origin preclude a claim that combined PD-L1, CD47, and IL-6 blockade is ready for IPF. Macrophages, T cells, and fibroblasts interact bidirectionally: immune signals such as TGF-β, IL-11, PDGF, and lipid mediators alter fibroblast states, whereas fibroblasts influence immune localization through chemokines, ECM barriers, and antigen-presentation programs. Defining causal order will require spatial proximity analysis, temporal sampling, and receptor–ligand perturbation.

Fibroblasts communicate through the ECM and extracellular vesicles as well as through soluble factors. The ECM is not merely structural; its composition, stiffness, and topography form a signaling interface that regulates epithelial and immune cells. ECM from IPF lung tissue promotes fibroblast activation and α-SMA expression [92], and increased stiffness activates YAP signaling and fibrotic and senescence programs, creating a self-reinforcing loop [54].

Extracellular vesicles add another layer of intercellular communication. In one experimental study, EVs from IPF fibroblasts were enriched in miR-23b-3p and miR-494–3p; uptake by alveolar epithelial cells suppressed SIRT3 and was associated with mitochondrial injury, senescence, and fibrosis [93]. In preclinical models, mesenchymal stromal cell-derived EVs delivered antifibrotic molecules such as miR-29c and miR-129 and promoted myofibroblast dedifferentiation and fibrosis resolution [53]. EVs therefore provide a mechanism for reciprocal regulation among fibroblasts, epithelial cells, and immune cells, although delivery, cargo specificity, and clinical translation remain unresolved [94,95].

### 5.2. Epithelial–Mesenchymal Interactions

Activated fibroblasts alter the epithelial microenvironment through secreted factors, EVs, and ECM. IPF fibroblast-derived EVs can induce mitochondrial injury and senescence in alveolar epithelial cells in vitro [93], and abnormal ECM composition and stiffness can impair the epithelial barrier and repair [92,96]. Experimental paracrine axes such as SPARC-EGFR-RAS suggest self-reinforcing epithelial–mesenchymal signaling [97,98]. However, the contributions of these mechanisms at different disease stages, and their necessity in human IPF, remain uncertain; co-culture alone cannot establish irreversible clinical progression.

Impaired AEC2 regeneration is a central feature of IPF. Normally, AEC2 cells proliferate and differentiate into alveolar type I epithelial cells to restore the barrier [99]; in IPF, senescence, abnormal autophagy, and impaired differentiation are observed [100,101]. SASP and other epithelial signals can activate fibroblasts, while fibroblasts impair epithelial repair through stiff ECM, SPARC, IL-11, and other mediators [44,93,98,102]. NR2F2 downregulation is associated with epithelial senescence and more severe experimental fibrosis [103]. These findings support abnormal epithelial–mesenchymal crosstalk in disease maintenance but do not prove that any single circuit inevitably causes irreversible progression. The dominant interaction may also differ by disease stage.

### 5.3. Immune–Mesenchymal Interactions

Macrophages are key regulators of fibroblast state [104], but the traditional M1/M2 classification poorly captures the macrophage states observed in human lung. IPF studies and single-cell analyses of silicosis have identified disease-associated states, including SPP1-high macrophages, with communication programs involving TGF-β, PDGF, galectin-3, and SPP1 [105,106]. In the bleomycin model, macrophage-derived exosomes transfer AT1R and enhance TGF-β/Smad signaling [107]. SPP1, TREM2, and CCR2 states overlap, and their origins may include resident macrophage transitions and monocyte recruitment. Together, these data support possible immune–mesenchymal feedback, but its causal role in human IPF requires spatial, temporal, and cell-specific validation. cGAS-STING may provide an additional injury-associated inflammatory input, although the available fluvoxamine evidence is preclinical and does not establish a necessary role in human IPF [82].

Fibroblasts reciprocally shape the immune microenvironment. ICAM-1, VCAM-1, CCL2, and CXCL12 can influence the recruitment and localization of macrophages, granulocytes, lymphocytes, and plasma cells [108,109]. Human IPF studies have linked specific mural-cell and fibroblast states to pathogenic plasma-cell niches, but the necessity and therapeutic tractability of this relationship remain uncertain [109]. Co-culture can reveal bidirectional communication but lacks intact tissue architecture, circulation, and mechanical context [110,111]. A self-amplifying loop therefore remains a working hypothesis until supported by functional blockade; co-expression of a ligand and its receptor is not sufficient evidence of a causal circuit.

**Figure 5:**
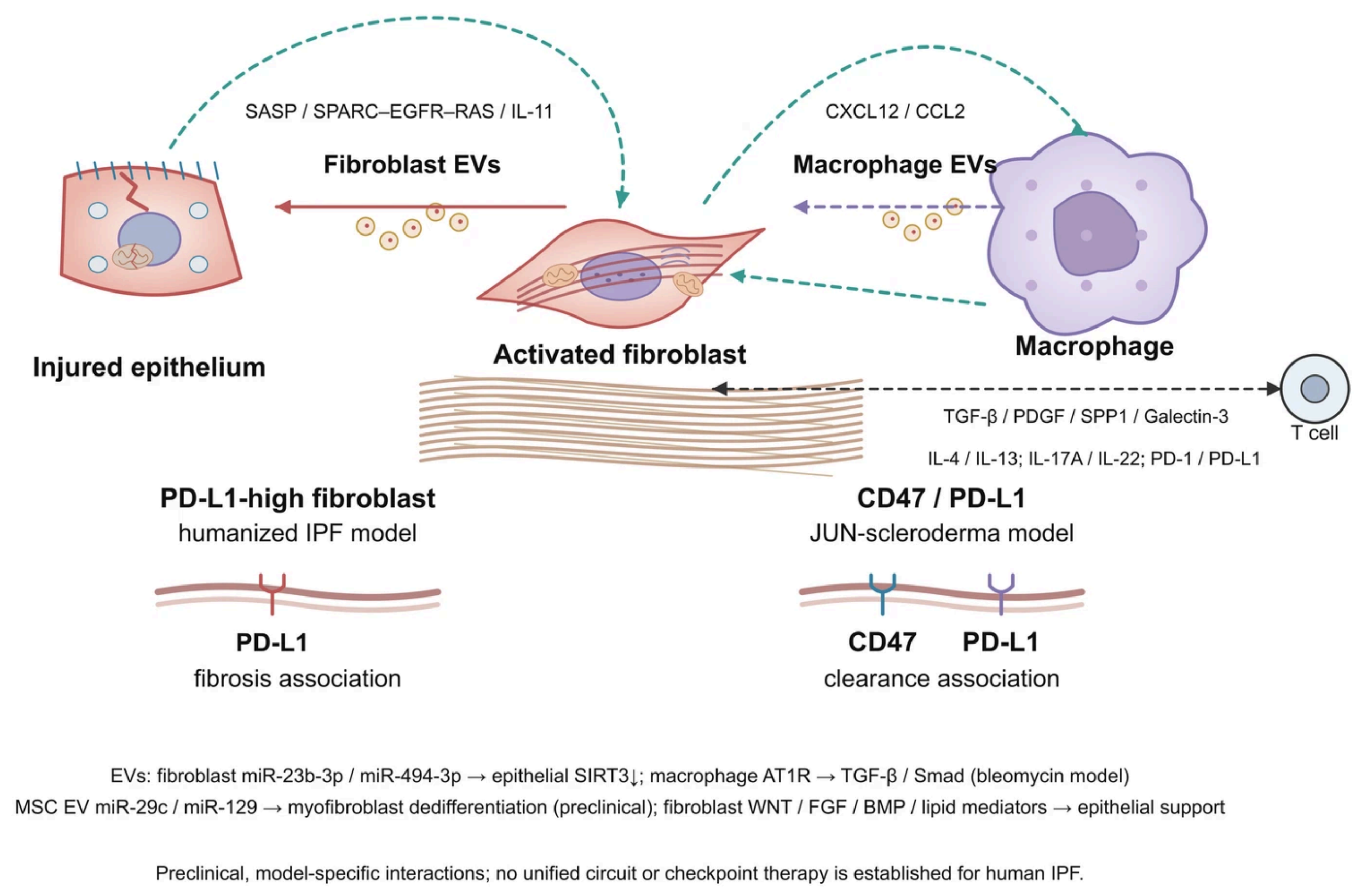
Representative epithelial-immune-fibroblast interactions involving morphology, EV transfer, and paracrine signaling. Arrow labels identify epithelial SASP/SPARC-EGFR-RAS/IL-11 signaling, fibroblast-derived CXCL12/CCL2, and macrophage-derived TGF-β/PDGF/SPP1/galectin-3. T-cell cytokines and PD-1/PD-L1 interactions are shown as context-dependent. The effects of fibroblast EV miR-23b-3p/miR-494–3p on epithelial SIRT3 and macrophage EV AT1R-TGF-β/Smad signaling are model-specific. MSC EV miR-29c/miR-129 and fibroblast WNT/FGF/BMP/lipid support are shown separately. The lower panels distinguish PD-L1-high fibroblasts in a humanized IPF model from CD47/PD-L1-associated clearance in a JUN-driven scleroderma model. These findings do not establish a unified causal circuit or checkpoint-directed therapy for human IPF. Red arrows indicate fibroblast extracellular-vesicle effects; purple dashed arrows indicate macrophage extracellular-vesicle effects. Green dashed arrows indicate intercellular signals. The black double-headed arrow indicates reciprocal fibroblast–T-cell communication.

Adaptive immune effects on mesenchymal cells are highly context-dependent. IL-4 and IL-13 can promote fibroblast activation directly or indirectly, but the roles of CD4^+^ LAG3^+^ T cells and TGF-β3 in SSc-ILD cannot be extrapolated uncritically to IPF [108,112]. In animal models, IL-17A and IL-22 can have opposing effects or show stage-dependent activity [113,114]. T-cell states are therefore neither uniformly profibrotic nor uniformly antifibrotic. Future studies should define disease type, compartment, stimulus, and sampling time and should test whether candidate immune signals directly and necessarily regulate fibroblast transitions. Figure 5 summarizes selected interactions discussed in this section.

## 6. Epigenetic and Metabolic Reprogramming

### 6.1. Epigenetic Regulation

DNA methylation changes in IPF are locus- and cell-type-specific and should not be described as universal hypermethylation of profibrotic promoters [115,116]. In experimental fibrosis, PPARγ promoter hypermethylation suppresses PPARγ expression and promotes fibrosis [117], and MBD2 and MeCP2 may regulate fibroblast activation through genes such as Erdr1 and WIF1 [118,119]. PM2.5 exposure is associated with altered methylation [120], but this association alone does not establish PM2.5 as an independent disease driver. DNA methyltransferases and methylation readers are candidate research targets, although tissue selectivity and long-term safety remain unresolved [121,122]. Methylation may repress antifibrotic genes, but it may also change secondarily during state transitions; histone acetylation and methylation, together with chromatin remodelers, can alter enhancer accessibility and modulate TGF-β, Wnt, and mechanosignaling outputs. Because many studies use whole lung or cultured cells, cellular composition and culture selection may confound the findings. Functional experiments are needed to determine which changes are causal. Future work should combine cell-type and spatially resolved epigenomics with gene editing or pharmacological perturbation to test locus-specific necessity [123,124,125,126,127].

Histone modifications contribute to fibrotic transcriptional programs, but current evidence points to model- and cell-type-dependent regulation rather than a single IPF epigenetic lock. KMT2A can increase PU.1-associated H3K4me3 and promote pulmonary fibrosis [127], whereas stiffness-associated HDAC3 loss and increased H3K9 acetylation can enhance COL1A1 and ACTA2 transcription [124]. The RCN3-EZH2-TGFBR1 circuit has also been linked to fibroblast activation [124]. These observations derive from different cell types, models, and endpoints and do not yet define a common human mechanism. Testing whether epigenetic changes constitute stable memory will require evidence that they persist after removal of the stimulus and are functionally required, as shown by locus- or enzyme-specific intervention. 

Noncoding RNAs regulate fibrotic transcription and signaling, but their effects depend on cellular origin and mechanism. miR-21 and the miR-29 family have been implicated in Smad feedback and collagen transcript regulation, respectively [125,126,128,129,130]; H19 loss attenuates bleomycin-induced inflammation and fibrosis, and PFI/SRSF1-related lncRNAs may have protective effects [131,132]. Circular RNAs, alternative splicing, alternative polyadenylation, and RNA modifications can further alter transcripts encoding receptors, metabolic enzymes, and ECM proteins. Most evidence remains from cell or animal models, and expression correlation alone cannot place an ncRNA upstream in a pathogenic pathway. Candidate ncRNAs require validation of cellular origin, subcellular localization, direct targets, dose–response, and in vivo delivery before they can be considered mechanistic nodes, biomarkers, or therapeutic vehicles. 

### 6.2. Metabolic Reprogramming

Enhanced glycolysis occurs in some IPF fibroblasts, but this phenotype varies with cell origin, stimulus, and disease stage and is not universal among pathological cells. It has been observed in primary human IPF cells, in vitro stimulation systems, and animal models. In vitro, TGF-β increases glycolytic enzymes such as PFKFB3 and LDHA through PI3K-Akt-mTORC1/HIF-1α [45], whereas a crystalline silica model implicates an IL-11-ERK-HIF-1α axis in glycolysis [46]. CBX5 is associated with glycolytic and mitochondrial balance, but its inhibition remains preclinical [31]. Glycolysis rapidly supplies ATP and supports nucleotide, amino acid, and lipid synthesis through the pentose phosphate pathway, serine synthesis, and acetyl-CoA production. Lactate accumulation and intracellular pH changes may also modulate TGF-β activation and epigenetic enzymes. Oxidative phosphorylation does not necessarily decrease in parallel, and some subsets may increase mitochondrial respiration or shift substrate use. Metabolic states should therefore be interpreted within defined subpopulations and under controlled oxygen tension and matrix stiffness [47,48,49,50,133,134,135,136].

HK2, PFKFB3, PKM2, and LDHA are altered in some human samples, in vitro systems, and animal models; additional axes include integrin β3-PKM2, circHIPK3/miR-30a-3p/FOXK2, and CPSF6-LDHA [47,48,49]. Inhibition of glycolysis attenuates profibrotic phenotypes in several preclinical models; anlotinib, for example, may act through PCBP3-PFKFB3 [50]. However, these drugs often have multiple targets, and normal tissues depend on the same metabolic pathways. Current evidence is therefore insufficient to establish glycolysis inhibition as a durable therapeutic mechanism in humans.

Glutamine metabolism and mitochondrial function may jointly support profibrotic responses, but most evidence remains preclinical. Experimental data show that TGF-β upregulates SLC1A5 and GLS1 and that glutamine-derived α-ketoglutarate can influence IPF fibroblast phenotype through metabolic and histone-modifying effects [51,52]. Because α-ketoglutarate supplementation rescues only part of the phenotype, both dependent and independent mechanisms are likely. IPF samples and models also show altered mitochondrial biogenesis, dynamics, ROS, and mitophagy [137,138,139,140]; however, tool compounds such as Mdivi-1 lack specificity, so animal efficacy cannot be attributed to a single mitochondrial target. Glutamine supplies carbon and nitrogen for proliferation and collagen synthesis, whereas α-ketoglutarate links the TCA cycle to dioxygenase-dependent epigenetic regulation. Altered fission, membrane potential, ROS, and mitophagy can change stress thresholds and promote a senescence-associated secretory phenotype. Evidence for GLS1, SLC1A5, or mitochondrial inhibition remains largely preclinical, and systemic toxicity plus dependence of normal repair cells on these pathways must be evaluated.

Lactate, methylmalonate, and alanine pathways have been linked to fibrotic phenotypes at different levels of evidence, but whether they sustain the human IPF fibroblast state remains unknown. Lactate can promote profibrotic responses through pH-dependent TGF-β activation and Akt2-PDK1 signaling [133,134]; a proposed role in collagen cross-linking still lacks direct evidence. The association between methylmalonate and TGF-β/Smad activation comes mainly from human samples, mouse studies, and cell work [136], whereas evidence for the GPT2/SLC38A2-alanine pathway derives from preclinical systems, including primary cells and human lung slices [141]. These observations identify testable targets but do not establish necessity in human IPF. Metabolite effects depend on concentration, cellular origin, and clearance: lactate is both a glycolytic product and a carbon source, altered fatty-acid oxidation may affect membrane lipids, redox balance, and ECM homeostasis, and age-related metabolites may modify immune, epithelial, and fibroblast compartments. Isotope tracing and spatial metabolomics are needed to distinguish altered flux from endpoint abundance and to avoid mistaking associated metabolites for drivers [133,134,135,136,141].

**Figure 6:**
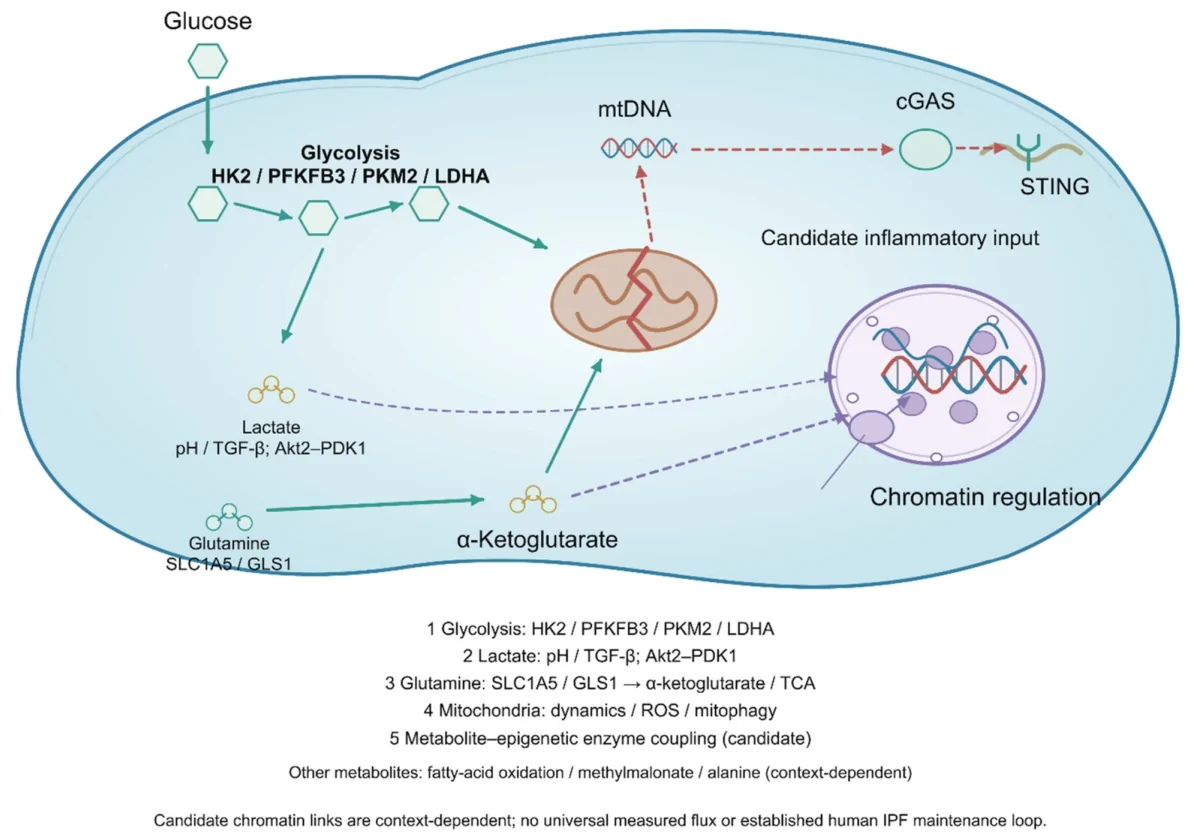
Metabolic–chromatin relationships in lung fibroblasts. The diagram depicts glycolysis, the glutamine/SLC1A5/GLS1–α-ketoglutarate–TCA axis, mitochondrial dysfunction, ROS and mitophagy, lactate signaling, and candidate links between metabolites and epigenetic enzymes. Additional metabolites are context-dependent examples. Dashed chromatin and mtDNA-cGAS-STING links indicate hypotheses, not universally measured fluxes or an established human IPF maintenance loop. Arrowheads show the indicated metabolic connections. Dashed links to chromatin and mtDNA–cGAS–STING denote proposed relationships.

Current evidence supports metabolic reprogramming in some profibrotic fibroblast states, but not a single metabolic pattern common to all pathological cells. Whether cGAS-STING connects mitochondrial injury to persistent fibroblast signaling within the proposed feedback layer remains testable and is not established in human IPF [82]. These findings should be interpreted alongside the shared model and analytical limitations in Section 7.2. Future studies should combine stable-isotope tracing, spatial metabolomics, and cell-specific perturbation to distinguish altered flux from endpoint abundance and true pathogenic dependence, while assessing effects on epithelial repair and immune function. Figure 6 summarizes the proposed links among epigenetic regulation, metabolism, and signaling.

### 6.3. A Two-Layer Stability Model of Epigenetic Maintenance and Signaling Feedback

**Figure 7:**
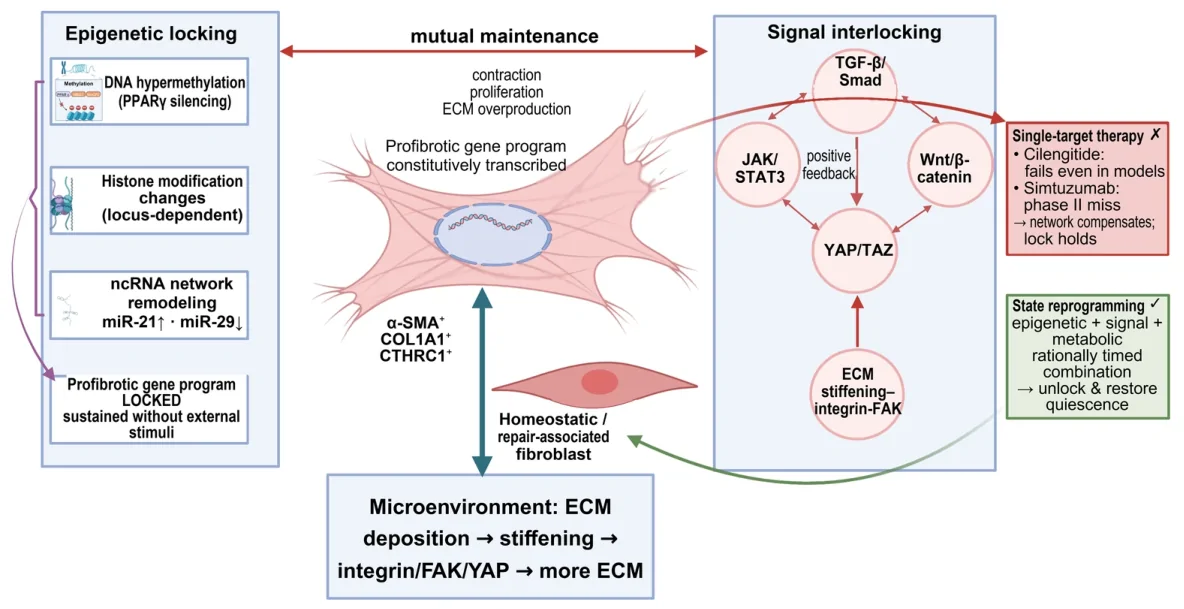
Working model for persistence of the profibrotic state. Histone changes are locus-, cell- and model-dependent; no uniform H3K4me3-increase/H3K27me3-decrease pattern is implied [124,127]. The lower cell represents a homeostatic/repair-associated state, not a universal PI16^+^CD82^+^ population [3,4,25,26]. Reciprocal maintenance, locking, reversion and timed combination treatment remain hypotheses. Molecular-label evidence: DNA hypermethylation/PPARγ silencing [117]; MBD2 and MeCP2 [118,119]; miR-21 increase/miR-29 decrease in experimental settings [125,126,128,129,130]; α-SMA, COL1A1 and CTHRC1 [3,4]; TGF-β/Smad [58], Wnt/β-catenin [69], JAK/STAT3 [77,78] and YAP/TAZ [74,142]; ECM–integrin–FAK signaling [70,72,76]; and model-specific cilengitide and phase II simtuzumab findings [143,144]. Epigenetic, signaling and metabolic co-targeting is proposed rather than clinically established [30,31,52]. The miniature methylation marks are schematic [115]. Red arrows depict signaling feedback; the green arrow depicts proposed state reprogramming. Purple arrows link epigenetic regulation to the persistent gene program. Blue arrows link cellular state and matrix feedback. The cross and check mark contrast single-target treatment limitations with proposed combined reprogramming; they do not establish clinical efficacy.

Persistence of the profibrotic state (Section 3.4) can be considered at two interacting levels. The first is epigenetic maintenance, involving chromatin accessibility, DNA methylation, histone modifications, and noncoding RNAs. The second is signaling feedback, involving TGF-β/Smad, Wnt/β-catenin, YAP/TAZ, and ECM-mediated mechanical input. We therefore propose a testable two-layer epigenetic maintenance–signaling feedback model (Figure 7), not an established disease mechanism. Its value lies in examining these mechanisms after removal of the initiating stimulus and in distinguishing induction, maintenance, and reversal. The model does not assume that either layer is necessary or sufficient, and it allows their relative contributions to vary across fibroblast subsets and disease stages. Individual chromatin regulators and matrix–signaling components have direct experimental support [31,38,39,40,75,76,124,127], and human tissue and chromatin data provide indirect support [30]; joint maintenance by both layers remains speculative. Compared with a generic positive-feedback loop or the epigenetic lock hypothesis, the model explicitly links both layers to post-stimulus persistence and predicts how intervention order should affect durability.

The model generates at least three falsifiable predictions. First, if epigenetic maintenance raises the threshold for signal inhibition, epigenetic modulation followed by signal inhibition should produce more durable reversal after drug withdrawal than either monotherapy or concurrent treatment once the profibrotic state is established; a lack of difference among these schedules would weaken the model. A sequential schedule of HDAC inhibition followed by YAP/TAZ inhibition provides a direct test of this prediction. Second, if ECM feedback is required for maintenance, replacing or softening the matrix while holding the intrinsic cellular state constant should produce sustained reductions in YAP/TAZ, Smad output, and ECM synthesis; transient changes would indicate that ECM feedback alone is insufficient. Third, if intracellular memory exists, some cells should retain reproducible chromatin, metabolic, and functional phenotypes after removal of stimuli such as TGF-β, and these phenotypes should be reversed by defined epigenetic perturbations. Lineage tracing, time-resolved single-cell multi-omics, long-term post-withdrawal assessment, and combinatorial genetic perturbation can distinguish these possibilities.

The model can be tested through three complementary experiments. First, activate primary human IPF fibroblasts for 5–7 days, match exposure across treatment schedules, and measure collagen output and contraction after 7–14 drug-free days. Second, transfer matched activated cells between matrices of controlled stiffness and measure nuclear YAP/TAZ, Smad output, and ECM production during treatment and after withdrawal. Third, pulse-label resident fibroblasts before bleomycin injury, examine established fibrosis and resolution, and combine chromatin readouts with cell-specific perturbation and post-withdrawal functional assessment. These are proposed protocols, not validated procedures.

## 7. Fibroblast Research in Pulmonary Fibrosis Models

Animal models are essential for dissecting fibrosis mechanisms and evaluating antifibrotic drugs, but each model captures different aspects of disease. The model should therefore be matched to the specific question about fibroblast activation, subset dynamics, or therapeutic targeting, and that choice defines the limits of extrapolation.

### 7.1. Common Models and Their Limitations

The bleomycin model reproduces inflammation, fibroblast activation, and ECM deposition and is widely used for mechanistic and pharmacodynamic studies. Injury progresses rapidly, however, and fibrosis resolves spontaneously in some protocols; the model therefore does not reproduce chronic IPF. The resolution window can nevertheless be used to study cellular and molecular mechanisms of fibrosis resolution [145].

Dosing timing strongly affects the interpretation of drug effects. Prophylactic dosing may primarily suppress inflammation and does not demonstrate treatment of established fibrosis, which can overestimate clinical antifibrotic potential. Cilengitide inhibited cell adhesion and collagen-related responses in vitro but showed no antifibrotic effect in the corresponding mouse study [143]. In bleomycin-treated hamsters, HJP272 given 1 h before injury reduced fibrotic outcomes, whereas dosing 24 h afterward was ineffective or only marginally effective; this early-treatment comparison did not test established fibrosis [146]. Thus, in vitro effects cannot be directly extrapolated to in vivo efficacy, but a single negative result is also insufficient to attribute failure to multifaceted locking.

The model has relatively high reproducibility and a well-defined time window, facilitating intervention comparisons, but inflammatory and fibrotic peaks vary with route, dose, animal age, and strain. To evaluate antifibrotic capacity, treatment should begin after ECM formation, and histologic, biochemical, mechanical, and pulmonary-function endpoints should be reported together.

Lineage tracing can test whether specific cells directly give rise to pathological matrix-producing cells, but conclusions are constrained by labeling efficiency, driver-gene specificity, and observation time. In a bleomycin mouse model, Rock et al. found no evidence that labeled AEC2 cells or airway epithelial cells formed myofibroblasts, nor did they support conversion of the pericyte populations examined into α-SMA-high cells [17]. This argues against a substantial direct EMT contribution in that model but cannot be used to derive a quantitative conclusion of ‘less than 5%’ in human IPF. Pleural mesothelial cells and pulmonary fibroblasts may participate in subpleural fibrosis through TGF-β-related signaling [147], but their contribution to human disease still requires direct evidence.

The limitations of single-dose bleomycin can be addressed with senescence-associated, persistent-signaling, repeated-injury, or human xenotransplantation models, depending on the question. AEC2 senescence models can examine how epithelial injury and SASP promote fibrosis [148], whereas xenotransplantation of human fibroblasts or IPF tissue preserves some human-cell features [149]. These models differ in drivers, time scale, and immune context; a single indirect reference cannot substantiate TGF-β1 transgenic, telomere-deficient, and humanized models simultaneously.

Xenotransplantation is further constrained by immunodeficiency, a nonphysiological transplant environment, and short observation periods, so extrapolation to chronic IPF requires explicit boundaries; it should not be described as having the greatest translational value. Repeated bleomycin, silica, and other persistent-injury models prolong fibrosis but introduce distinct immune and toxicological backgrounds. Human lung slices, organoids, air–liquid interface co-cultures, and decellularized ECM systems retain selected features of the human microenvironment and are useful for species comparisons, but they lack intact circulation, a complete immune system, and long-term mechanical loading. They should complement rather than replace in vivo models.

### 7.2. Model Selection and Evidence Extrapolation

Model selection should follow the research question. Bleomycin is suitable for post-injury inflammation and early fibrosis and for initial pharmacodynamic assessment. Persistent transgenic or senescence-associated models can address chronic signaling and host susceptibility. Human fibroblast xenotransplantation can assess human-cell responses, although immunodeficiency limits study of immune–stromal interactions [149]. No single model reproduces the age-related context, spatial heterogeneity, chronic progression, and treatment response of IPF, so key conclusions should be validated in at least two complementary models and in human tissue. Dissociation, sampling, batch effects, and clustering resolution can all alter apparent populations. Trajectory inference remains hypothesis-generating, and cross-study comparisons require the species, disease stage, and functional validation described in Section 2.3.

## 8. Therapeutic Strategies Targeting Fibroblast Heterogeneity and Functional States

Fibroblasts are important effectors in antifibrotic therapy, yet current drugs were not developed for specific fibroblast subsets. Signaling pathways, metabolism, epigenetic maintenance, and intercellular communication provide candidate intervention points, while local delivery, state-selective treatment, and mechanism-guided combinations are emerging directions (Table 2). Most candidates remain preclinical or early clinical, and they should be distinguished from approved antifibrotic drugs, which are not subset-specific.

### 8.1. Targeting Signaling Pathways and the Microenvironment

TGF-β/Smad signaling is central to fibroblast activation, but TGF-β also regulates immunity and tissue homeostasis; prolonged broad inhibition therefore carries safety risks. Receptor inhibitors, Smad7 enhancement, and local delivery remain preclinical or proof-of-concept strategies and are not established IPF therapies. Wnt/β-catenin inhibitors such as ICG-001 and HSP90 inhibitors show antifibrotic activity in cells and animals [64,69]. Because Wnt signaling also supports alveolar epithelial repair, translation will require concurrent assessment of cell selectivity and long-term safety.

FAK and YAP/TAZ connect matrix stiffness to fibroblast state, but their therapeutic value remains largely proof of concept. Triptolide can modulate MMP/LOX/integrin-related ECM remodeling, and dopamine D1 receptor agonists may inhibit YAP/TAZ through cAMP-dependent signaling and improve experimental fibrosis [142,150]. These findings support targeting ECM–cell feedback, but the drugs are not fibroblast-selective, and dose, tissue exposure, and systemic toxicity limit translation. Labeling a mechanosignaling pathway as a key node does not establish that its isolated blockade will produce durable benefit in IPF.

Approved IPF therapies include nintedanib, pirfenidone, and nerandomilast (Jascayd), all of which slow disease progression rather than target specific fibroblast subsets. Nintedanib inhibits PDGFR, FGFR, VEGFR, and related kinases, whereas pirfenidone has broad antifibrotic effects [154,155]. Nerandomilast preferentially inhibits PDE4B and is administered orally. In FIBRONEER-IPF, adjusted mean changes in FVC at week 52 were −114.7 mL with 18 mg twice daily, −138.6 mL with 9 mg twice daily, and −183.5 mL with placebo; differences from placebo were 68.8 mL and 44.9 mL, respectively [156]. The US FDA approved nerandomilast for adults with IPF in October 2025 and for progressive pulmonary fibrosis in December 2025 [157]; geographic approval status and indications should be verified separately. None of these drugs is subset-specific, and slowed progression does not demonstrate reversal of established scarring.

Rentosertib, a TNIK inhibitor, provides an exploratory phase IIa signal rather than established efficacy. The compound was discovered with generative-AI assistance; its 71-participant, 12-week phase IIa study primarily assessed safety and tolerability. FVC increased from baseline by 98.4 mL in the 60 mg once-daily group and decreased by 20.3 mL in the placebo group, but the study was not powered for efficacy and did not show a stable monotonic dose–response relationship [152]. This FVC difference is therefore hypothesis-generating and cannot confirm clinical benefit. The phase III GENESIS-IPF-3 study (NCT07687459) has annual FVC decline over 52 weeks as its primary endpoint [153]; long-term safety and incremental benefit over background antifibrotic therapy remain to be demonstrated. TNIK remains a candidate convergence node, but the proposed simultaneous blockade of Wnt, TGF-β, and YAP requires direct validation.

Targeting intercellular communication or the ECM may complement pathway inhibition, but model benefit has not translated reliably into clinical benefit. Integrin subtypes differ in ligands, cellular sources, and roles in TGF-β activation, and the failure of an αvβ6 antibody illustrates the risk of direct extrapolation [72,158]. IL-11 neutralization and CXCL12/CXCR4 inhibition show activity in preclinical models [43,44,109], but neither has demonstrated clinical efficacy. LOXL2 inhibition had a mechanistic rationale, yet simtuzumab showed no clinical benefit [144,159]. Collagenase and MMP-modulating strategies should therefore remain experimental and should not be presented as treatments capable of directly reversing established human scarring.

### 8.2. Targeting Fibroblast Subpopulations

The FAP-targeted CAR-T concept was first validated in cardiac fibrosis [161]. In a bleomycin mouse model, in vivo LNP-mRNA delivery produced transient FAP CAR-T cells, reduced FAP^+^-activated stromal cells, and improved histology and alveolar-cell states [160]. FAP is a membrane target suitable for delivery or cell therapy, whereas CTHRC1 is primarily a marker of a pathological ECM-associated state; they are not equivalent cell-surface targets [3]. Human efficacy, target coverage, off-target injury, and long-term effects on repair remain unknown. Transient CAR-T expression may reduce toxicity from persistent activity, but the approach remains preclinical. Antibody–drug conjugates, local nucleic-acid delivery, and microenvironment-activated drugs will each need separate evidence of tissue access and safety.

Another strategy is to protect or restore homeostatic fibroblast functions. Meflin^+^ fibroblasts limit scar formation in experimental fibrosis [5], whereas PDGFRA^+^ lipofibroblasts support the alveolar niche and repair but may become profibrotic after injury; they cannot be classified simply as protective [4,27]. No clinically validated Meflin agonist or PDGFRA^+^-selective strategy currently exists. Mesenchymal stromal cell-derived EVs can deliver miR-29c, miR-129, and other cargo and promote fibrosis resolution in models [53], but manufacturing, tissue distribution, and batch consistency remain major challenges. A shift from subset elimination to state modulation is biologically plausible and testable, but current evidence does not show superiority to existing therapy.

### 8.3. Targeting Metabolism and Epigenetic Regulation

State modulation aims to reduce profibrotic activity while preserving repair function, rather than simply eliminating cells. PFKFB3- and PKM2-dependent glycolysis, SLC1A5/GLS1-dependent glutamine metabolism, and macropinocytosis are altered in some activated fibroblasts and fibrosis models, and their inhibition can attenuate profibrotic phenotypes [50,51,52,162]. Evidence for macropinocytosis currently comes from cells, explants, and animals, with no clinical data [162]. These pathways also support normal cell function, and current data do not show that their inhibition can selectively and durably restore a physiological state. True reprogramming should therefore be assessed through cell fate, ECM turnover, and tissue function after drug withdrawal, with nonspecific cytotoxicity excluded.

Epigenetic interventions may weaken maintenance of profibrotic programs, but evidence remains preclinical. DNMT, MBD2/MeCP2, HDAC, and KMT2A inhibitors show antifibrotic activity in cell or animal models [118,119,124,127]. Because these enzymes have broad physiological roles, such findings do not establish that the drugs inevitably release an epigenetic lock. H19-related and PFI/SRSF1-related noncoding-RNA strategies differ in functional direction, cellular origin, and delivery requirements [131,132]. Stable state reversal will require evidence that a target persists in pathological human IPF cells, can be engaged with tissue selectivity, and retains its effect after withdrawal; transient transcriptional suppression alone is insufficient.

Metabolites can regulate epigenetic enzymes, and epigenetic states can regulate metabolic genes and receptors, creating a potential bidirectional relationship [52]. However, a metabolism–epigenetics positive-feedback loop has not been established in a defined fibroblast state. Combination studies should test monotherapy, concurrent treatment, sequential treatment, and post-withdrawal outcomes while measuring target engagement, toxicity, epithelial repair, and ECM function. Mechanistic compatibility does not imply pharmacological synergy; formal dose–response and interaction analyses remain necessary.

### 8.4. Rationale for Combination Interventions and Evidence Gaps

**Figure 8:**
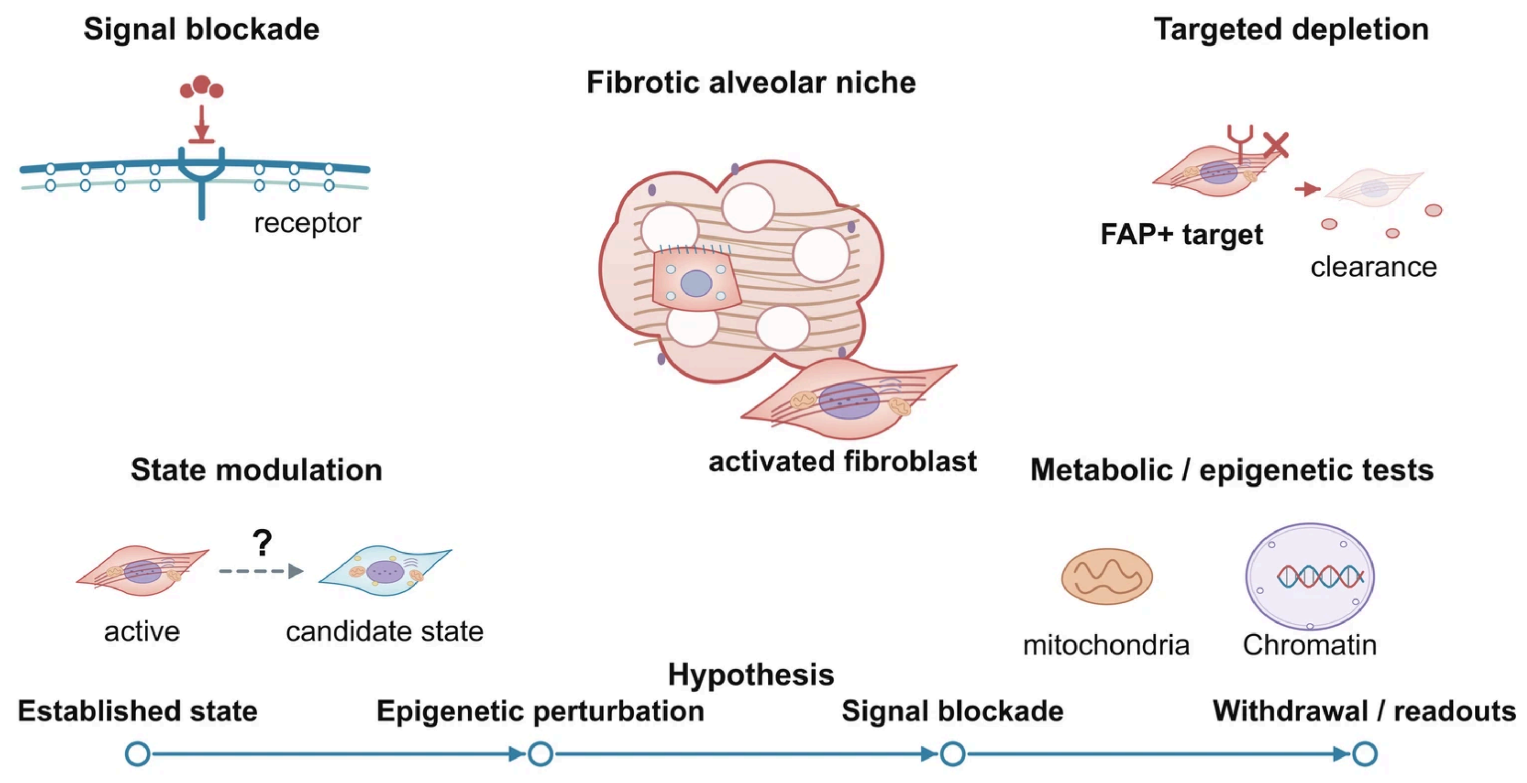
Therapeutic strategies organized around a fibrotic alveolar niche. Five callouts illustrate intervention classes, with selected Table 2 examples and data-row references (header excluded). The targets are examples, not an exhaustive list. Pairings in Table 3 are hypotheses rather than established combinations. The timeline includes monotherapy, concurrent treatment, forward and reverse sequencing, and post-withdrawal readouts. No statement in this figure implies clinical scar reversal, an optimal sequence, or proven combination efficacy. The blue timeline arrows indicate the proposed treatment sequence. The dashed state-change arrow and question mark indicate a candidate transition requiring validation.

Combination interventions may be justified by pathway redundancy, metabolic support, epigenetic maintenance, and ECM feedback, but most proposed pairs have not been tested directly. Candidate approaches include epigenetic modulation plus signal inhibition, metabolic inhibition plus pathway blockade, and pathogenic-state restriction combined with repair support. Studies should include monotherapy, concurrent, and sequential controls; confirm tissue exposure and target engagement; and evaluate toxicity, epithelial repair, and post-withdrawal stability. The combinations in Table 3 are intended only to test the two-layer model. No approved IPF therapy includes a Meflin agonist or SPP1 blocker, and these pairings are not clinical recommendations. If a combination is no better than monotherapy or loses effect after withdrawal, the assumptions of pathway complementarity or temporal synergy should be reconsidered. Figure 8 groups therapeutic strategies by intervention logic.

## 9. Challenges in Clinical Translation and Future Directions

### 9.1. Translational Gap Between Preclinical Models and Clinical Efficacy

Preclinical efficacy does not predict success in a randomized IPF trial. Translational failure can result from poor alignment between model and human disease, prophylactic dosing, inadequate tissue exposure, a target that is not necessary for disease maintenance, toxicity-limited dosing, patient heterogeneity, or an insensitive endpoint. Simtuzumab, the αvβ6 antibody BG00011, pamrevlumab in ZEPHYRUS-1, and SAR156597 all failed in clinical testing [144,158,163,164]. Their failure mechanisms are not interchangeable, and none justifies attributing all trial failure to fibroblast epigenetic locking. More informative development will require therapeutic dosing after fibrosis is established, use of complementary models, confirmation of target expression and exposure in human lesions, and pharmacodynamic endpoints in early trials.

### 9.2. Patient Stratification and Candidate Biomarkers

Clinical heterogeneity in IPF reflects fibroblast state, epithelial injury, immune context, genetic susceptibility, and disease stage; it cannot be reduced to fibroblast subset composition. SPP1-related immune signals, ECM features, circulating EVs, and FAP imaging are candidate stratification or pharmacodynamic biomarkers, but none has completed the analytical validation, clinical validation, and multicenter replication required for routine use. In explanted lungs from advanced ILD, FAPI-PET uptake correlated with immunohistochemistry [165], providing human tissue evidence that still requires prospective validation for stratification. Tissue measurements such as H3K4me3/H3K27me3 are also affected by sampling site and cellular composition. A rigorous path forward would use a prospective cohort with standardized processing and prespecified thresholds, followed by independent validation to establish incremental predictive value for progression or treatment response and cross-center reproducibility.

Clinical translation of fibroblast-targeted therapies must address models, targets, delivery, and biomarkers together. Models must represent established fibrosis and chronic progression; target studies must demonstrate expression in human lesions, functional necessity, and adequate tissue exposure; delivery studies must evaluate intrapulmonary distribution, systemic toxicity, and effects on normal repair cells; and biomarker studies must complete source confirmation, assay validation, and independent replication. Only when these components are integrated can a negative trial be distinguished from wrong-target, inadequate-exposure, enrollment, or endpoint failure, and only then can a positive mechanistic signal become a reproducible clinical benefit.

## 10. Conclusions

Pulmonary fibroblast heterogeneity includes both regional differences among anatomical subsets and continuous injury-induced state transitions. Single-cell and spatial studies show that pathological states such as CTHRC1^+^ fibroblasts accumulate in fibrotic lesions, but their lineage origins, stability, and causal contribution to human disease remain unresolved. Heterogeneity and functional remodeling are dynamically linked: anatomical context constrains the initial response, whereas injury-induced states can cross regional boundaries and reshape the local niche. Research should therefore move beyond static cell inventories to examine the origin, duration, functional necessity, persistence, and reversibility of fibroblast states.

The profibrotic state may be maintained by interacting TGF-β/Smad, Wnt/β-catenin, YAP/TAZ, and ECM mechanical feedback, with epigenetic and metabolic changes raising the threshold for reversal. The two-layer epigenetic maintenance-signaling feedback model provides a framework for integrating these observations, but its necessity, sufficiency, and optimal intervention sequence require experimental validation.

Therapeutic design should inhibit pathogenic signals or states while preserving normal repair. FAP, CTHRC1, YAP/TAZ, and PFKFB3 are supported by different levels of evidence and are not yet validated clinical targets. Future work should prioritize human-tissue target validation, therapeutic rather than prophylactic study designs, and post-withdrawal functional and tissue-stability endpoints to determine whether state reprogramming is real and durable.

## Figures and Tables

**Table 1:** Molecular features and functional localization of major fibroblast subpopulations in healthy and fibrotic lung tissue.

Role and Population	Representative Markers	Species, Evidence, Niche and Role	Refs.
Anatomical/homeostatic identity—Perialveolar fibroblasts	SCUBE2, INMT, FGFR4	Human/mouse atlas evidence; support alveolar structure and the epithelial niche in alveolar septa. Marker validation varies by study.	[3]
Anatomical/homeostatic identity—Peribronchial fibroblasts	COL15A1, ENTPD1, LGR5	Human/mouse atlas evidence; support airways and stabilize the basement membrane in the subepithelial airway region. Marker validation is study-specific.	[3]
Anatomical/homeostatic identity—Vascular adventitial fibroblasts	PI16, COL15A1	Human/mouse evidence; markers vary by species and study. Support perivascular matrix and mechanics in the vascular adventitia.	[3,23]
Anatomical/homeostatic identity—Alveolar duct fibroblasts	Low ACTA2; high PI16	Primarily mouse; location-defined state at the alveolar–airway junction that supports transitional-zone structure.	[3]
Homeostatic/repair-associated—Quiescent lipofibroblasts	ADIPOQ, PI16, CD82, CD36, CD97	Lipid storage and repair regulation in alveolar regions. PI16: mouse single-cell/spatial evidence and human lung-slice functional testing; CD82: human single-cell, tissue and flow-cytometric evidence; CD36/CD97: immunofluorescence/flow cytometry in primary human fibroblasts and IPF tissue. VCAM1/CD106 is excluded: mesothelial-associated in cited human data and low/nonspecific in mouse fibroblast datasets.	[4,24,25]
Disease-associated state—CTHRC1^+^ pathological fibroblasts	CTHRC1, COL1A1; ACTA2 variable	Human/mouse; associated with fibrotic lesions. High-volume collagen synthesis and profibrotic activity in fibroblast foci.	[3]
Disease-associated state—WNT5A^+^CTHRC1^+^ subpopulation	WNT5A, CTHRC1	Human IPF spatial/single-cell studies; disease-associated state enriched in dense fibrotic regions. Functional and therapeutic significance needs further validation.	[22]
Disease-associated state—Activated lipofibroblasts	ADIPOQ, PI16	Primarily mouse models; invasion, proliferation and matrix deposition in injury/alveolar regions.	[4]
Disease-associated state—Ebf1^+^ fibroblasts	EBF1, CD55	Primarily mouse models; matrix remodeling and enhanced contraction in injury regions.	[4]
Disease-associated state—Inflammatory proliferative fibroblasts	GREM1, PPP2R3A	Mouse silica model with human IPF expression and in vitro confirmation; associated with inflammatory responses in early lesions.	[6]
Disease-associated state—Senescence-associated fibroblasts	p16, p21, SASP	Human primary lung fibroblasts; DNA-damage-induced senescence and human IPF expression. Associated with SASP/ECM imbalance; not an independent lineage in fibrotic regions.	[26]
Candidate homeostatic/repair state—Meflin^+^ fibroblasts	MEFLIN	Human IPF tissue, mouse bleomycin model and fibroblast experiments; limits scar formation in experimental models. Normal interstitium.	[5]
Homeostatic/repair-associated—PDGFRA^+^ lipofibroblasts	PDGFRA, ADIPOQ	Human/mouse; supports alveolar niche and repair but can enter a profibrotic state after injury. Function is state-dependent.	[4,27]

Note: SASP, senescence-associated secretory phenotype.

**Table 2:** Overview of therapeutic strategies targeting pulmonary fibroblast heterogeneity and functional remodeling.

Section/Target	Strategy and Intended Action	Development Stage	Evidence, Limitations and Refs.
8.1 Signaling—TGF-β/Smad	Receptor inhibitors or Smad7 enhancement to inhibit myofibroblast differentiation and collagen synthesis.	Preclinical/conceptual	Broad inhibition may disrupt immunity and tissue homeostasis; subtype-selective or local delivery is needed. Ref. [42]
8.1 Signaling—Wnt/β-catenin	ICG-001 or HSP90 inhibitors (e.g., ganetespib) to inhibit matrix remodeling and support epithelial repair.	Preclinical	Safety evidence is insufficient; Wnt also contributes to alveolar epithelial repair. Refs. [64,69]
8.1 Signaling—FAK/YAP/TAZ	Triptolide (ECM/LOX/integrin axis) or dopamine D1 agonists (YAP/TAZ) to weaken matrix–cell feedback.	Preclinical	Proof-of-concept evidence; these agents are not fibroblast-selective, and tissue exposure and systemic toxicity remain concerns. Refs. [148,149]
8.1 Signaling—JAK/STAT	Baricitinib or ruxolitinib to inhibit inflammatory–fibrotic signaling.	Preclinical/related-disease evidence	Evidence for ILD indications is limited; drug effects in related diseases do not establish efficacy in IPF. Refs. [77,78,79,80,150]
8.1 Signaling—TNIK	Rentosertib; proposed convergence-node inhibition.	Phase IIa exploratory; Phase III registered	The 71-participant, 12-week Phase IIa study was not powered for efficacy and showed no stable monotonic dose–response; Phase III confirmation is pending. Refs. [151,152]
Approved baseline—PDGFR/FGFR/VEGFR	Nintedanib; inhibit kinases involved in fibroblast proliferation, migration and differentiation.	Approved	Slows progression but is not specific to fibroblast subpopulations. Ref. [153]
Approved baseline—TGF-β/Smad and collagen genes	Pirfenidone; broad antifibrotic effects that reduce collagen/ECM expression.	Approved	Not specific to fibroblast subpopulations. Ref. [154]
Approved baseline—PDE4 (preferentially PDE4B)	Nerandomilast (Jascayd); slows FVC decline with antifibrotic and immunomodulatory effects.	Approved in the US for IPF and PPF (as of 20 September 2026)	Not specific to fibroblast subpopulations; long-term real-world data remain limited. Refs. [155,156,157]
8.1 Microenvironment—Integrins (β1, αvβ6)	ATN-161 or αvβ6 inhibitors to inhibit pathological adhesion and TGF-β activation.	Preclinical; αvβ6 clinical failure	Integrin targets are not interchangeable; αvβ6 clinical failure does not establish effects of other integrin strategies. Refs. [72,158]
8.1 Microenvironment—IL-11	IL-11-neutralizing antibody to reduce fibroblast–epithelial/immune communication.	Preclinical	Antifibrotic activity is reported in cell and animal studies; efficacy in humans is unestablished. Refs. [43,44]
8.1 Microenvironment—CXCL12/CXCR4	Chemokine-axis antagonists to disrupt a profibrotic immune–stromal niche.	Preclinical/translational exploration	The axis is associated with a pathological niche; therapeutic efficacy is unproven. Ref. [109]
8.1 Microenvironment—LOXL2/ECM	LOXL2 inhibitors to reduce matrix cross-linking and stiffness-driven feedback.	Phase II clinical failure	Simtuzumab showed no clinical benefit; other ECM-degradation strategies require direct validation. Refs. [159,160]
8.2 Subpopulations—FAP/CTHRC1	Antibody–drug conjugate or transient FAP CAR-T to target FAP^+^ pathological mesenchymal cells.	Preclinical	CTHRC1 is not an equivalent cell-surface entry point. Human efficacy, off-target effects, repair effects and long-term safety remain unclear. Refs. [3,161,162]
8.2 Subpopulations—Meflin/PDGFRA	Restore Meflin function or modulate PDGFRA^+^ cells; no validated agonist is available.	Preclinical	Activation mechanisms and tractability in human IPF are unclear; PDGFRA^+^ cells can become profibrotic after injury. Refs. [5,25]
8.2 Subpopulations—MSC/EVs	EV delivery associated with miR-29c/miR-129 to promote paracrine myofibroblast dedifferentiation.	Preclinical	Delivery, tissue distribution and manufacturing standardization remain limitations. Ref. [53]
8.3 Metabolic—Glycolysis	PFKFB3/HK2 inhibitors or anlotinib to restrict energy and biosynthetic substrates.	Preclinical	Effects on normal-cell metabolism and tissue repair require assessment. Refs. [47,50]
8.3 Metabolic—Glutamine metabolism	Transporter/glutaminase blockade to reduce profibrotic activity.	Preclinical	Whether inhibition produces stable state reversion remains unvalidated. Refs. [51,52]
8.3 Metabolic—Macropinocytosis	Macropinocytosis inhibitors to attenuate profibrotic phenotypes.	Preclinical	Peer-reviewed evidence includes cells, explants and animal models; no clinical data are available. Ref. [163]
8.3 Epigenetic—DNA methylation	DNMT inhibitors or MBD2/MeCP2 targeting to regulate methylation-related programs.	Preclinical	Tissue selectivity and long-term safety data are lacking. Refs. [118,119]
8.3 Epigenetic—Histone modifications	HDAC or KMT2A inhibitors to modulate chromatin state and profibrotic transcription.	Preclinical	Effects cannot be assumed to be inevitably reversible. Refs. [124,127]
8.3 Epigenetic—Noncoding RNA	H19-related or PFI/SRSF1-related strategies to modulate post-transcriptional regulation.	Preclinical/conceptual	Delivery, cell specificity and off-target effects remain unresolved. Refs. [131,132]

Note: HDAC, histone deacetylase;CAR-T, chimeric antigen receptor T cell; MSC, mesenchymal stromal cell; FAP, fibroblast activation protein. Preclinical evidence includes cellular, explant, and animal studies and does not imply efficacy in humans. Drug approval status and indications are current as of 20 September 2026.

**Table 3:** Examples of combination interventions proposed for validation of the two-layer stability model.

Combination Proposal	Empirical Support in This Review and Evidence Gap	Minimal Test and Outcome That Would Weaken the Rationale
Epigenetic modulation → YAP/TAZ inhibition	Chromatin regulators and matrix–signaling components have individual experimental support [31,38,39,40,75,76,124,127]. The cited evidence does not establish sequence superiority or durable reversal for this pair; joint maintenance remains speculative.	After a profibrotic state is established, compare monotherapy, concurrent treatment, and the epigenetic-to-signal sequence. Measure collagen output during treatment and after withdrawal. A lack of post-withdrawal advantage over monotherapy or concurrent treatment would not support the prediction.
PFKFB3 inhibitor + TGF-β receptor inhibitor	Both targets are discussed as preclinical intervention points [42,47,50]. The review does not cite a direct test of this pair or show that metabolic restriction sustains signal inhibition.	Compare each monotherapy with concurrent and sequential treatment in a relevant experimental model. Assess profibrotic activity, epithelial repair, and ECM function during treatment and after withdrawal. No added benefit, or loss of benefit after withdrawal, would weaken the rationale.
FAP-directed targeting + candidate Meflin restoration	FAP-directed strategies remain preclinical [161,162], and Meflin protection is experimental [5]. No validated Meflin agonist or direct combination evidence is cited; this remains a concept.	Once a Meflin-restoring intervention is defined, compare each intervention alone with the combination. Measure matrix burden and epithelial repair; a lack of added benefit or impaired repair would argue against this pairing.
IL-11 neutralization + SPP1 inhibition	IL-11 neutralization has cell/animal support [43,44], but the review cites no direct evidence for this pair and states that SPP1 blockers are not approved IPF drugs. Clinical benefit is not established.	Compare each intervention with the combination in a model that captures immune–mesenchymal interactions. Assess fibroblast activation, immune-cell recruitment, toxicity, and epithelial repair. A lack of added effect or impaired repair would weaken the rationale.

Note: IL-11, interleukin 11; TAZ, transcriptional co-activator with PDZ-binding motif.

## Data Availability

No new data were created or analyzed in this study. Data sharing is not applicable to this article.
